# Effect of Benign Biopsy Findings on an Artificial Intelligence–Based Cancer Detector in Screening Mammography: Retrospective Case-Control Study

**DOI:** 10.2196/48123

**Published:** 2023-08-31

**Authors:** Athanasios Zouzos, Aleksandra Milovanovic, Karin Dembrower, Fredrik Strand

**Affiliations:** 1 Department of Oncology and Pathology Karolinska Institute Stockholm Sweden

**Keywords:** artificial intelligence, AI, mammography, breast cancer, benign biopsy, screening, cancer screening, diagnostic, radiology, detection system

## Abstract

**Background:**

Artificial intelligence (AI)–based cancer detectors (CAD) for mammography are starting to be used for breast cancer screening in radiology departments. It is important to understand how AI CAD systems react to benign lesions, especially those that have been subjected to biopsy.

**Objective:**

Our goal was to corroborate the hypothesis that women with previous benign biopsy and cytology assessments would subsequently present increased AI CAD abnormality scores even though they remained healthy.

**Methods:**

This is a retrospective study applying a commercial AI CAD system (Insight MMG, version 1.1.4.3; Lunit Inc) to a cancer-enriched mammography screening data set of 10,889 women (median age 56, range 40-74 years). The AI CAD generated a continuous prediction score for tumor suspicion between 0.00 and 1.00, where 1.00 represented the highest level of suspicion. A binary read (flagged or not flagged) was defined on the basis of a predetermined cutoff threshold (0.40). The flagged median and proportion of AI scores were calculated for women who were healthy, those who had a benign biopsy finding, and those who were diagnosed with breast cancer. For women with a benign biopsy finding, the interval between mammography and the biopsy was used for stratification of AI scores. The effect of increasing age was examined using subgroup analysis and regression modeling.

**Results:**

Of a total of 10,889 women, 234 had a benign biopsy finding before or after screening. The proportions of flagged healthy women were 3.5%, 11%, and 84% for healthy women without a benign biopsy finding, those with a benign biopsy finding, and women with breast cancer, respectively (*P*<.001). For the 8307 women with complete information, radiologist 1, radiologist 2, and the AI CAD system flagged 8.5%, 6.8%, and 8.5% of examinations of women who had a prior benign biopsy finding. The AI score correlated only with increasing age of the women in the cancer group (*P*=.01).

**Conclusions:**

Compared to healthy women without a biopsy, the examined AI CAD system flagged a much larger proportion of women who had or would have a benign biopsy finding based on a radiologist’s decision. However, the flagging rate was not higher than that for radiologists. Further research should be focused on training the AI CAD system taking prior biopsy information into account.

## Introduction

Breast cancer is the most common cancer among women worldwide. It ranks fifth as a cause of cancer deaths because of its relatively favorable prognosis, but in the last 20 years, the average annual increase in breast cancer incidence rate has been 1.4% [1-3]. Screening programs have been clearly proven to reduce the mortality rate for breast cancer [4-6]. Retrospective studies have shown that outcomes might improve when radiologists combine mammography readings with an artificial intelligence (AI) system for computer-aided detection (CAD) [7-9]. Furthermore, reducing reading time with the assistance of an AI CAD system is possible [10,11]. An AI CAD system can be highly accurate for reading mammograms, and some systems are now on a comparable level with average breast radiologists at detecting breast cancer on screening mammography [12].

In addition to the well-known risk factors of age, family history, and hormonal history, there are also studies showing that benign breast disease increases the risk of breast cancer [13,14]. A study that analyzed risk factors for breast cancer found that having undergone any prior breast procedure was associated with an increased risk of breast cancer [15]. Another study showed that women found to have false-positive mammography findings were more likely to develop interval cancer or cancer at the second screening compared to those not recalled [16].

Radiologists performing screen reading normally have access to information about prior biopsies, while AI CAD systems do not take this information into account. In this retrospective study, we analyzed primarily to what extent the malignancy assessments of an AI CAD system are affected by the presence or absence of biopsy-proven benign findings. In a secondary analysis, we determined whether this effect differs between an AI CAD system and radiologists.

## Methods

### Study Population 

This retrospective study was based on a case-control subset from the Cohort of Screen-Aged Women (CSAW). The CSAW is a complete population-based cohort of women aged 40 to 74 years invited to screening in the Stockholm region, Sweden, between 2008 and 2015 [17]. The exclusion criteria in the CSAW were having a prior history of breast cancer, having a diagnosis outside the screening range, and having had incomplete mammographic examinations. From the CSAW, a case-control subset was separately defined to contain all women from Karolinska University Hospital, Stockholm, who were diagnosed with breast cancer (n=1303), those at screening or clinical evaluation during the interval before the next planned screening, and 10,000 randomly selected healthy controls [17]. The purpose of the case-control subset is to make evaluation more efficient by not having to process an unnecessary amount of healthy controls while preserving the representability of the CSAW screening cohort in which it is nested. Additional exclusion criteria for the current study were having implants and receiving a cancer diagnosis later than 12 months after mammography. The study population was divided into 3 groups based on their status: cancer, benign biopsy, and normal.

The cancer group was defined as having biopsy-verified breast cancer at screening or within 12 months of screening. The most recent mammographic screening prior to diagnosis was selected for analysis. The benign biopsy group was defined as having had a benign biopsy finding without ever having had breast cancer. The group was further stratified by the interval between biopsy and mammography. The normal group had neither breast cancer nor a prior benign biopsy finding. Women in the screening program who were previously recalled and deemed as having benign disease were also included in this group.

### Mammography Assessments

The screening system consisted of double-reading followed by consensus discussion for any flagged examination. The following screening decision data were collected: flagging of abnormal screening by one or both radiologists and the final recall decision after consensus discussion. Screening decisions and clinical outcome data were collected by linking to regional cancer center registers.

### AI CAD system

The AI CAD system was an Insight MMG (version 1.1.4.3; Lunit Inc). The reason for choosing Insight MMG for this study was that it demonstrated superior results in a retrospective analysis published in 2020 [9], which compared 3 AI CAD systems with a sensitivity and specificity comparable to Breast Cancer Surveillance Consortium benchmarks [18]. Briefly, the AI CAD system was originally trained on 170,230 mammograms from 36,468 women diagnosed with breast cancer and 133,762 healthy controls. The AI CAD system had been validated by previous studies using a deep learning model to triage screening mammograms [11,19]. The mammograms in the original training set were sourced from 5 institutions: 3 from South Korea, 1 from the United States, and 1 from the United Kingdom. The mammograms were acquired on mammography equipment from GE Healthcare, Hologic, and Siemens, and there were both screening and diagnostic mammograms. The generated prediction score for tumor presence was a decimal number between 0.00 and 1.00, where 1.00 represented the highest level of suspicion. The program assessed 2 images of each breast, and the highest score among the 4 images was selected to represent the examination. To obtain a binary assessment, determining whether the examination should be considered flagged for further workup by the AI CAD system, a cutoff point is required, above which the examination is considered flagged and below which the examination is considered not flagged. The cutoff point (0.40; AI abnormality threshold) defined whether an examination was considered flagged or not flagged by an AI CAD system, and was predefined in a prior study [9]. The cutoff was selected to enforce that the specificity of the AI CAD system should be the same as that for the average radiologist in that study. The examinations in the prior study originated from the same institution and are partly overlapping, which should ensure that the cutoff value is transferrable to the current setting.

### Data Collection

The Stockholm-Gotland Regional Cancer Center provided personal identification numbers for all women who fulfilled the inclusion criteria for the CSAW. The identification numbers were linked to the local breast cancer quality register, “Regional Cancercentrum Stockholm-Gotlands Kvalitetsregister för Bröstcancer,” to collect data about breast cancer diagnosis. All diagnoses of breast cancer were biopsy verified. Benign diagnoses were collected from hospital electronic health records. All images were 2D full-field digital mammograms acquired on Hologic mammography equipment. The personal identification numbers were also linked to the radiological image repository to extract all digital mammograms from the Picture Archiving and Communication System.

### Statistical Analysis

Statistical analysis was performed per patient and not per lesion. Stata (version 14 or later; StataCorp) was used for statistical analyses. The Wilcoxon rank-sum test and quantile regression analysis were used to examine differences between groups. To perform statistical tests, differences in medians were chosen due to the skewed distribution of AI scores. The required level for statistical significance was not adjusted for multiple comparisons. A value of *P*<.05 was considered statistically significant.

### Ethics Approval

The collection and use of the data set by AI was approved by the Swedish Ethical Review Board (2017-02-08), and the need for informed consent was waived (diary number 2016/2600-31).

## Results

**Figure 1:**
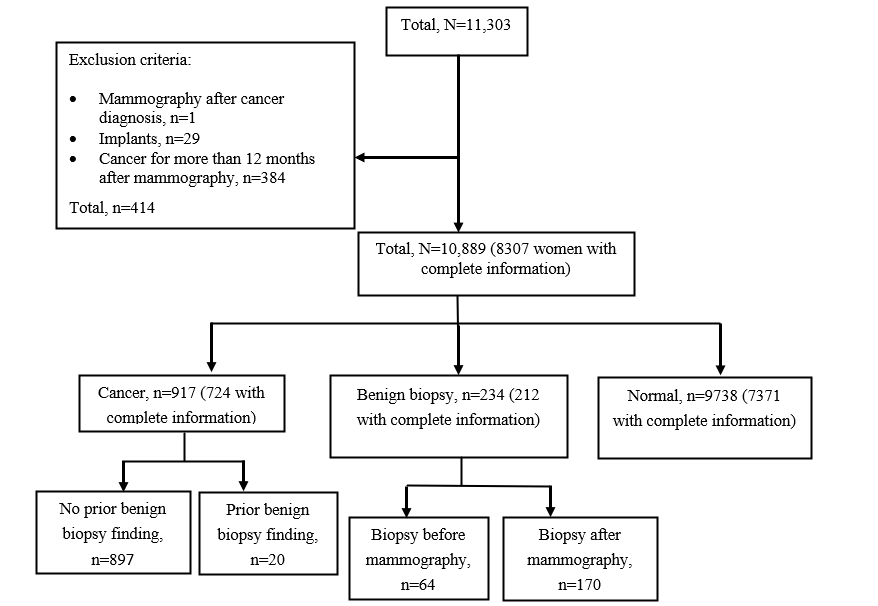
Study population with exclusion criteria and subgroups.

We evaluated 11,303 women for inclusion in this retrospective case-control study (Figure 1). Of them, 414 women were excluded. The exclusion criteria were no mammographic examination in conjunction with a cancer diagnosis, having implants, and having cancer more than 12 months after mammography. The cancer group consisted of a total of 917 women, the benign biopsy group comprised 234 women, and the group with no cancer or biopsy (control group) comprised 9738 women.

Of the remaining 10,889 women, 8269 had complete information regarding radiologist assessments (when performing data collection, we received radiologist assessments only until December 31, 2015), which included selections rendered as potentially pathological by 1 or both radiologists and a final recall decision after consensus discussion. From those 8269 women, there were 724 women in the cancer group, 212 in the benign biopsy group, and 7371 in the normal mammography group.

There was a significant difference (*P*<.001) in AI scores among the cancer, benign biopsy, and normal mammography groups (Table 1).

**Table 1:** Characteristics of the study population.

Characteristics	Participants, n	Age (years), median (IQR)	Proportion of assessments above the cutoff point, % (n/n)	AI^a^ score, median (IQR)	*P* value (comparison with the cancer group)	*P* value (comparison with the normal biopsy group)
Normal with benign biopsy	234	51.2 (45.6-53.0)	11 (26/234)	0.051 (0.016-0.174)	<.001	<.001
Cancer	917	60.6 (50.7-66.6)	83 (768/917)	0.933 (0.666-0.983)	N/A^b^	<.001
Normal without biopsy	9738	55.5 (48.8-65.2)	3.5 (345/9738)	0.018 (0.005-0.065)	<.001	N/A

^a^AI: artificial intelligence.

^b^N/A: not applicable.

The proportion of AI assessments above the cutoff point was 3.5% in the group with normal mammography findings and 83% in the cancer group. In the benign biopsy group, 11% of the AI assessments were above the cutoff point. The distribution of AI scores for women diagnosed with breast cancer is shown in Multimedia Appendix 1, that for healthy women with a benign biopsy in Multimedia Appendix 2, and that for healthy women without a benign biopsy who remained healthy in Multimedia Appendix 3.

In Table 2, we show how the AI score is associated with the age of the women. There was a significant increase of the AI score in relation to age category in the cancer group (*P*<.05). There was no significant increase in the AI score in relation to age in the group with normal mammographic findings or in the group with benign biopsy findings. The median age for the study population was 56 years, and the median AI score was 0.023. The median age for the cancer group was 61 years, and the median AI score was 0.933 (*P*=.01). The median age of the group with previous benign biopsies was 49 years, and the median AI score was 0.051 (*P*=.71). The median AI score for healthy women was 0.018 (*P*=.40), and the median age of that group was 59 years.

The benign biopsy group was stratified by the interval between biopsy and mammography into 3 categories: 0-6 months, 6-24 months, and more than 24 months. There was no significant difference among the time-stratified categories (Table 3). In Table 3, we describe the AI score related to the time between biopsy and mammography. Within 6 months after mammography, 104 of 234 participants had had a benign biopsy finding. The proportion of women with AI scores above the threshold was 16% for those with a benign biopsy finding within 6 months from mammography and 33% for those with a benign biopsy finding 6 months before mammography.

In the group with a benign biopsy finding after mammography, the proportion of abnormal assessments by AI, above cutoff point, was 15%, while the radiologists had a recall rate up to 57% for this group (Table 4). The radiologists had a recall rate of 2%, and the rate for abnormal assessments by AI was 3.8% in the group with normal mammograms and that with benign biopsy findings (Table 4). For the group with only normal mammograms, the recall rates were 1% and 3.6%, respectively. Radiologists and the AI program had similar rates of recall for the total study population.

**Figure 2:**
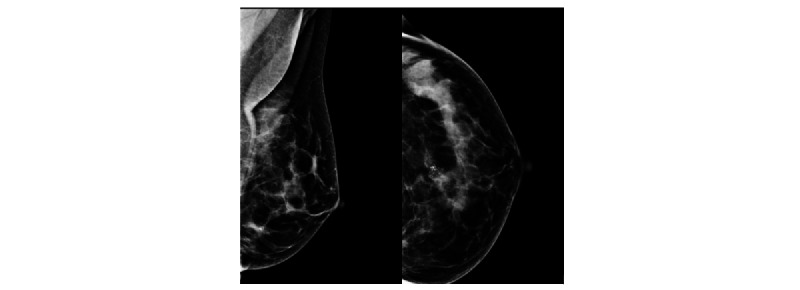
A 50-year-old woman selected by radiologists for potential pathology in the left breast. A high artificial intelligence (AI) score was assigned. The biopsy results showed hyperplastic breast epithelial cells that could represent a degenerated fibroadenoma.

**Figure 3:**
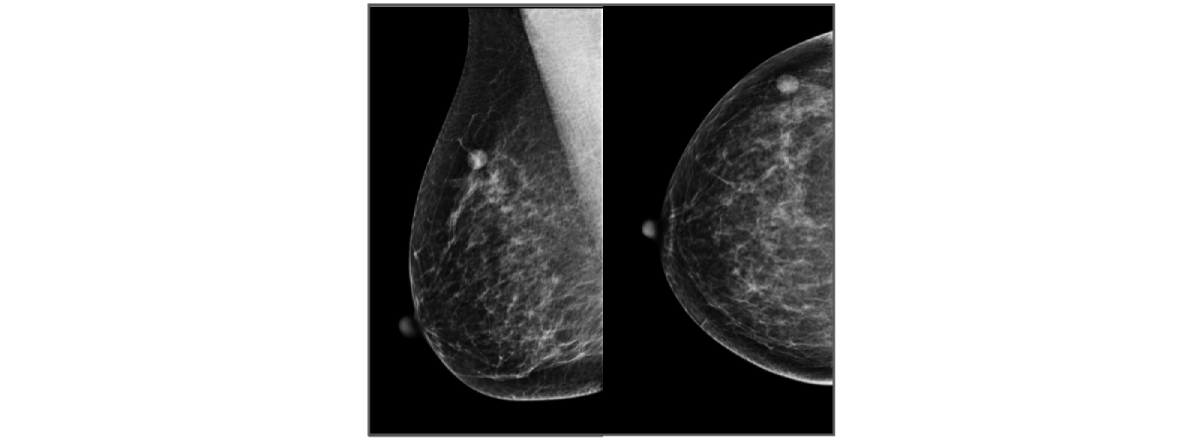
A 56-year-old woman selected by radiologists for potential pathology in the right breast. A low artificial intelligence (AI) score was assigned. The biopsy shows the lymph node.

The 2 screening mammograms shown in Figures 2 and 3 have been assessed by radiologists and the AI cancer detection program. These examples illustrate concordant and discordant assessments.

**Table 2:** Artificial intelligence (AI) score for each age group of the normal, benign biopsy, and cancer groups.

Age group (years)	Normal mammography			Benign biopsy group									Cancer group							
	All groups (*P*=.40)			All groups (*P*=.71)		Benign biopsy findings before mammography (*P*=.71)			Benign biopsy findings after mammography (*P*=.81)			All groups (*P*=.01)				Prior benign biopsy findings (*P*=.54)			No benign biopsy findings (*P*=.01)	
	Participants, n	AI score, median (IQR)	Participants, n		AI score, median (IQR)	Participants, n	AI score, median (IQR)	Participants, n		AI score, median (IQR)	Participants, n			AI score, median (IQR)	Participants, n		AI score, median (IQR)	Participants, n		AI score, median (IQR)
All	9738	0.018 (0.005-0.065)	234		0.051 (0.016-0.174)	64	0.069 (0.018-0.179)	170		0.047 (0.016-0.205)	917			0.933 (0.667-0.983)	20		0.924 (0.747-0.986)	897		0.934 (0.666-0.983)
40-49	3152	0.021 (0.006-0.068)	144		0.048 (0.016-0.155)	43	0.074 (0.019-0.142)	101		0.042 (0.012-0.176)	213			0.861 (0.272-0.974)	9		0.861 (0.286-0.974)	204		0.864 (0.264-0.975)
50-59	2834	0.015 (0.005-0.058)	68		0.051 (0.017-0.260)	16	0.039 (0.014-0.113)	52		0.051 (0.017-0.305)	228			0.948 (0.715-0.985)	3		0.911 (0.891-0.950)	225		0.949 (0.709-0.985)
60-69	2577	0.018 (0.005-0.064)	22		0.078 (0.012-0.174)	5	0.173 (0.090-0.449)	17		0.047 (0.011-0.144)	379			0.935 (0.723-0.984)	8		0.945 (0.887-0.991)	371		0.935 (0.720-0.984)
≥70	1175	0.020 (0.006-0.077)	0		N/A^a^	0	N/A	0		N/A	97			0.958 (0.820-0.985)	0		N/A	97		0.958 (0.820-0.985)

^a^N/A: not applicable.

**Table 3:** Mammographic examinations of women a benign biopsy finding having an artificial intelligence (AI) score above the predefined threshold for cancer suspicion, grouped by the timing of the biopsy.

Timing of biopsy		Participants, n	Age (years), median (IQR)	Proportion of assessments above the cutoff point, % (n/n)	AI score	*P* value
**Benign biopsy finding before mammography (months)**		64				
	0-6	9	49.1 (44.5-54.9)	33 (3/9)	0.150 (0.099-0.449)	Reference
	6-24	39	48.1 (46.7-52.7)	5.1 (2/39)	0.062 (0.016-0.150)	.12
	>24	16	41.0 (40.3-48.4)	0 (0)	0.025 (0.015-0.099)	.06
**Benign biopsy after mammography (months)**		170				
	0-6	104	48.3 (44.4-52.5)	16 (17/104)	0.065 (0.017-0.274)	Reference
	6-24	44	49.0 (45.6-55.3)	4.5 (2/44)	0.037 (0.008-0.109)	.36
	>24	22	51.2 (45.6-53.0)	9 (2/22)	0.031 (0.017-0.165)	.38

**Table 4:** Recall rate and abnormal assessments by artificial intelligence.

Assessments				Recall rate, % (n/n)						
				Radiologist 1		Radiologist 2		Consensus		Abnormal AI assessments above the cutoff point
Total				10 (831/8307)		10 (827/8307)		9.2 (767/8307)		11 (880/8307)
**Normal and benign biopsy findings**				3.6 (274/7583)		3.1 (234/7583)		2 (154/7583)		3.8 (290/7583)
	Normal			2.8 (203/7371)		2.2 (162/7371)		1 (73/7371)		3.6 (265/7371)
	**Benign biopsy findings**			33 (71/212)		34 (72/212)		38 (81/212)		12 (25/212)
		Biopsy before mammography	8.5 (5/59)		6.8 (4/59)		6.8 (4/59)		8.5 (5/59)	
		Biopsy after mammography	49 (66/135)		50 (68/135)		57 (77/135)		15 (20/135)	
**Cancer**				77 (557/724)		82 (593/724)		85 (613/724)		81 (590/724)
	With benign biopsy findings			75 (12/16)		88 (14/16)		88 (14/16)		81 (13/16)
	Without benign biopsy findings			77 (545/708)		82 (579/708)		85 (599/708)		82 (577/708)

## Discussion

The AI CAD system in this study showed increased flagging of screening examinations for women with benign biopsy findings compared to those for healthy women without biopsies. However, the flagging rate was similar between AI and radiologists for women with a prior biopsy finding, and considerably lower for women with a biopsy finding after screening.

For women with a previous benign biopsy finding, compared to healthy women, the AI CAD system’s flagging rate (false positives) increased from 3.6% to 8.5%. In other words, there was a significant difference in AI scores between the normal group and the benign biopsy group despite both groups consisting of women without breast cancer. This finding might raise questions about the probability that AI is affected by alternations on mammography because of the biopsy. This did not seem to be the case, since we found a similar increase in recall rate for the radiologists from 2.8% to 8.5%. This is unexpected since radiologists had access to the outcomes of prior biopsies while AI did not.

For women who had a benign biopsy finding after screening, we found that 57% of them resulted from recall by the screening radiologists. Applying the AI CAD system in screening would have resulted in a much lower false positive flagging rate of only 15% for the AI program. Based on this observation, one may suggest further research on the role of AI in reducing the number of unnecessary biopsies.

The strength of this study is the large number of women with cancer and that all women were sampled from a screening cohort. Another strength of this study is the use of the specific AI algorithm, which has already been validated in large cohorts with very positive results [9]. Our data of the total recall rates and specifically those of the cancer group amplify the indications from previous studies that AI-based cancer detectors can be reliable enough to be incorporated in a screening setting.

The main limitation is the relatively small number of benign biopsies, which makes it difficult to consider the effect of different types of benign lesions. Another limitation is the study’s retrospective setting. Since the AI program did not have the opportunity to make recalls and choose women for further diagnostic biopsy, it could not influence who received a biopsy after screening, and all decisions about benign biopsies were based on radiologists’ assessments. In contrast to radiologists, the AI program calculates a score for the likelihood of breast cancer based on the image alone and does not consider any information about symptoms given by the woman at screening.

Furthermore, in this study, we did not consider the exact location of the presumed abnormality where the AI program revealed a high AI score. Further analysis of the data can be valuable to evaluate whether the lesions that AI showed responded to the actual finding that the patient was recalled for.

In conclusion, the tested AI CAD system had an increased flagging rate of 8.5% for women with a prior benign biopsy finding; this rate was not higher than that for radiologists who often have information about prior benign biopsy findings. Further research and development might be focused on how to further improve AI CAD systems by taking into account information about prior benign biopsy findings.

## Appendix

Multimedia Appendix 1 Artificial intelligence (AI) score distribution for the cancer group (n=917).

Multimedia Appendix 2 Artificial intelligence (AI) score distribution for the benign biopsy group (n=234).

Multimedia Appendix 3 Artificial intelligence (AI) score distribution for the normal group (n=9738).

## Abbreviations

AI: artificial intelligence
CAD: computer-aided detection
CSAW: Cohort of Screen-Aged Women
RCC: Regional Cancer Center
