# Supplementary material for: Effect of Benign Biopsy Findings on an Artificial Intelligence–Based Cancer Detector in Screening Mammography: Retrospective Case-Control Study
Source: JMIR AI. 2023 Aug 31;2:e48123. doi: 10.2196/48123 (PMC11041399; doi:10.2196/48123)
Supplement: Multimedia Appendix 1 [file ai_v2i1e48123_app1.docx]

**Multimedia Appendix 1.** Artificial intelligence (AI) score distribution for the cancer group (n=917).
